# Development and validation of Nomograms for predicting overall survival and Cancer-specific survival in patients with ovarian clear cell carcinoma

**DOI:** 10.1186/s13048-020-00727-3

**Published:** 2020-10-17

**Authors:** Qian Chen, Shu Wang, Jing-He Lang

**Affiliations:** Department of Obstetrics and Gynecology, Peking Union Medical College Hospital, Chinese Academy of Medical Science and Peking Union Medical College, 1 ShuaiFuYuan, Wangfujing, DongCheng District, Beijing, 100730 P.R. China

**Keywords:** Ovarian clear cell carcinoma, Nomogram, Predict, Overall survival, Cancer-specific survival

## Abstract

**Background:**

Ovarian clear cell carcinoma (OCCC) is a rare histologic type of ovarian cancer. There is a lack of an efficient prognostic predictive tool for OCCC in clinical work. This study aimed to construct and validate nomograms for predicting the overall survival (OS) and cancer-specific survival (CSS) in patients with OCCC.

**Methods:**

Data of patients with primary diagnosed OCCC in the Surveillance, Epidemiology, and End Results (SEER) database between 2010 and 2016 was extracted. Prognostic factors were evaluated with LASSO Cox regression and multivariate Cox regression analysis, which were applied to construct nomograms. The performance of the nomogram models was assessed by the concordance index (C-index), calibration plots, decision curve analysis (DCA) and risk subgroup classification. The Kaplan-Meier curves were plotted to compare survival outcomes between subgroups.

**Results:**

A total of 1541 patients from SEER registries were randomly divided into a training cohort (*n* = 1079) and a validation cohort (*n* = 462). Age, laterality, stage, lymph node (LN) dissected, organ metastasis and chemotherapy were independently and significantly associated with OS, while laterality, stage, LN dissected, organ metastasis and chemotherapy were independent risk factors for CSS. Nomograms were developed for the prediction of 3- and 5-year OS and CSS. The C-indexes for OS and CSS were 0.802[95% confidence interval (CI) 0.773–0.831] and 0.802 (0.769–0.835), respectively, in the training cohort, while 0.746 (0.691–0.801) and 0.770 (0.721–0.819), respectively, in the validation cohort. Calibration plots illustrated favorable consistency between the nomogram predicted and actual survival. C-index and DCA curves also indicated better performance of nomogram than the AJCC staging system. Significant differences were observed in the survival curves of different risk subgroups.

**Conclusions:**

We have constructed predictive nomograms and a risk classification system to evaluate the OS and CSS of OCCC patients. They were validated to be of satisfactory predictive value, and could aid in future clinical practice.

## Introduction

Ovarian cancer (OC) is one of the most aggressive gynecological cancer, consisting of a group of heterogonous tumors. As a subtype of epithelial ovarian cancer (EOC), ovarian clear cell carcinoma (OCCC) presents a distinct biological profile from other histological types [1]. With a higher incidence in East Asia (~ 30%), OCCC is reported to be diagnosed at a younger age compared with serous carcinoma [2].

Patients with early-stage OCCC generally exhibit favorable prognosis, while those in advanced stage present worse survival outcomes than patients in the high-grade serous group [3]. In addition to stage, other factors were proposed to exert influences on the prognosis of OCCC, such as the presence of endometriosis, surgical methods and venous thromboembolism [4, 5]. Demographic characteristics, tumor size, lymph node status and treatment strategies are also important when evaluating survival outcomes.

Standard treatment guidelines for OCCC have not yet been developed, given its rarity in the large clinical trials of EOC. A comprehensive prognostic judgment system would be useful to guide the selection of a treatment protocol. The nomogram, a statistic-based predictive tool with the ability to integrate pivotal predictive factors, has been widely utilized to quantify risks and evaluate the prognosis of many cancer types [6–8]. However, to the best of our knowledge, no nomograms for patients with OCCC have been developed. In this study, we aimed to construct nomograms using data extracted from the Surveillance, Epidemiology, and End Results (SEER) database to predict the prognosis of patients with OCCC.

## Materials and methods

### Data source

Medical records of patients with OCCC were obtained from the SEER database, which contains data of cancer patients from 18 regional registries, covering approximately 34.6% of the total population in the United States [9]. Relevant information was extracted applying SEER*Stat software version 8.3.6 (https://seer.cancer.gov/seerstat/).

### Data extraction

Patients diagnosed as OCCC from 2010 to 2016 were selected through the International Classification of Diseases for Oncology, 3rd edition (ICD-O-3) morphology codes “8310/3; 8313/3; 8443/3; 8444/3” from OC patients. Variables for this study included age at diagnosis, race, laterality, grade, stage (American Joint Commission on Cancer [AJCC] 7th version), tumor size, organ metastasis, radiotherapy, chemotherapy, number of examined lymph nodes (LNs), LN status, vital status and survival time. The organ metastasis sites referred to liver, lung, bone, and brain according to the available data in the SEER database.

Patients with OCCC were excluded in the following scenarios: (1) not the primary tumor; (2) without histologic confirmation; (3) survival time shorter than 1 month; (4) no surgery; and (5) unknown information about LN, race, tumor size, stage and organ metastasis.

### Statistic methods

Patients enrolled in our study were randomly assigned into the training cohort and validation cohort at a ratio of 7:3. The primary end points were overall survival (OS) and cancer-specific survival (CSS). Categorical variables were shown as frequencies and proportions. The comparison of clinicopathological characteristics between the training and validation cohorts was performed using a chi-squared test.

The least absolute shrinkage and selection operator (LASSO) method was used to primarily select useful predictive features to avoid over-fitting to some extent. Significant prognostic factors were further identified in multivariate analysis from the Cox proportional hazards model. Then, the nomograms associated with OS and CSS were constructed incorporating the final risk factors.

The performance of the nomogram was validated internally in the training cohort and externally in the validation cohort. Harrell’s concordance index (C-index) ranging from 0.5 to 1.0 was used to evaluate the discriminative abilities of the nomograms. Calibration curves (1000 bootstrap resamples) were generated to test the consistency between the predicted and actual 3- and 5-year OS and CSS. Emerging as a new method, decision curve analysis (DCA) was applied to evaluate the latent value of the nomograms [10]. Moreover, the whole cohort was regrouped into low- and high-risk groups with the median risk score generated from the nomogram. Kaplan-Meier analysis and log-rank test were used to explore the survival difference between risk subgroups.

All statistical analyses were performed with SPSS (version 25.0, SPSS, Chicago, IL, USA) and R software (version 3.6.0; http://www.r-project.org/). A *P* value of < 0.05 was considered statistically significant.

## Results

### Patient characteristics and survival outcomes

A total of 1541 patients diagnosed with primary OCCC were identified from the SEER database. Most of the patients were at an early stage (78.3%) and the proportion of white patients (74.9%) was the greatest. 1223(79.4%) patients underwent lymph node dissection and 1265 (82.1%) received chemotherapy. Characteristics of patients in the training cohort (*n* = 1079) and the validation cohort (*n* = 462) were listed in Table 1.

**Table 1:** Patients’ demographics and clinicopathological characteristics

Variables	All patients n (%)	Training set n (%)	Validation set n (%)	P
Total	1541 (100.0)	1079 (70.0)	462 (30.0)	
Age, years				0.984
< 50	413 (26.8)	290 (26.9)	124 (26.8)	
50–60	609 (39.5)	425 (39.4)	184 (39.8)	
> 60	518 (33.6)	364 (33.7)	154 (33.3)	
Tumor size, cm				0.325
≤ 12	810 (52.6)	576 (53.4)	234 (50.6)	
> 12	731 (47.4)	503 (46.6)	228 (49.4)	
Race				0.524
White	1154 (74.9)	813 (75.3)	341 (73.8)	
Other	387 (25.1)	266 (24.7)	121 (26.2)	
Laterality				0.289
Unilateral	1374 (89.2)	968 (89.7)	406 (87.9)	
Bilateral	167 (10.8)	111 (10.3)	56 (12.1)	
AJCC Stage (7th)				0.730
I	979 (67.5)	687 (63.7)	292 (63.2)	
II	167 (10.8)	111 (10.3)	56 (12.1)	
III	302 (19.6)	214 (19.8)	88 (19.0)	
IV	395 (6.0)	67 (6.2)	26 (5.6)	
Grade				0.167
I/II	121 (7.9)	87 (8.1)	34 (7.4)	
III	588 (38.2)	429 (39.8)	159 (34.4)	
IV	398 (25.8)	266 (24.7)	132 (28.6)	
Unknown	434 (28.2)	297 (27.5)	137 (29.7)	
LN dissected, n				0.154
No	318 (20.6)	217 (20.1)	101 (21.9)	
1–10	446 (28.9)	328 (30.4)	118 (25.5)	
> 10	777 (50.4)	534 (49.5)	243 (52.6)	
LN				0.233
No dissection or Negative	1370 (88.9)	966 (89.5)	404 (87.4)	
Positive	171 (11.1)	113 (10.5)	58 (12.6)	
Organ Metastasis				0.939
No	1505 (97.7)	1054 (97.7)	451 (97.6)	
Yes	36 (2.3)	25 (2.3)	11 (2.4)	
Radiotherapy				0.312
No/Unknown	1509 (97.9)	1054 (97.7)	451 (98.5)	
Yes	32 (2.1)	25 (2.3)	7 (1.5)	
Chemotherapy				0.800
No/Unknown	276 (17.9)	195 (18.1)	81 (17.5)	
Yes	1265 (82.1)	884 (81.9)	381 (82.5)	

*AJCC* American Joint Committee on Cancer, *LN* lymph node

The 3- and 5-year OS rates were 77.8, and 70.6% for all patients, respectively, with a mean follow-up time of 65.3 months. The 3- and 5-year CSS rates were 77.8, and 70.6% for all patients, respectively, with a mean follow-up time of 66.8 months. The 3- and 5-year OS and CSS rates of all patients in terms of different clinical features were shown in Table 2.

**Table 2:** The 3-and 5-year overall survival and cancer-specific survival in terms of patient characteristics

Variables	Overall Survival		Cancer-Specific Survival	
	3-year (%)	5-year (%)	3-year (%)	5-year (%)
Age, years				
< 50	73.4	66.3	74.4	67.8
50–60	82.0	73.7	82.9	75.3
> 60	76.4	70.2	78.6	73.4
Tumor size, cm				
≤ 12	77.4	72.8	79.0	75.0
> 12	78.4	68.4	79.6	70.4
Race				
White	79.2	72.5	80.7	74.8
Black	57.7	40.3	61.3	42.8
Other	76.8	68.8	77.4	70.0
Laterality				
Unilateral	81.8	75.1	83.1	77.2
Bilateral	46.4	35.5	48.4	37.1
AJCC Stage (7th)				
I	91.2	85.6	92.2	87.2
II	76.9	74.5	78.3	–
III	46.7	34.1	49.0	35.8
IV	40.8	27.9	41.7	30.5
Grade				
I/II	85.4	73.6	86.3	79.2
III	75.9	67.4	77.0	69.2
IV	78.5	75.0	80.9	77.3
Unknown	–	–	–	–
LN dissected, n				
No	64.1	59.7	66.7	63.3
1–10	78.4	70.6	79.6	72.7
> 10	83.1	75.1	84.2	76.6
LN				
No dissection or Negative	78.5	70.7	79.9	72.6
Positive	72.5	69.8	74.3	70.1
Organ Metastasis				
No	79.0	71.8	80.5	73.9
Yes	30.5	22.2	30.5	26.7
Radiotherapy				
No/Unknown	78.0	70.7	79.5	72.9
Yes	66.4	33.2	66.4	–
Chemotherapy				
No/Unknown	80.5	76.1	83.4	80.5
Yes	77.2	69.4	78.4	71.1

*AJCC* American Joint Committee on Cancer, *LN* lymph node

### Construction of the prognostic nomograms for OS and CSS

**Fig. 1:**
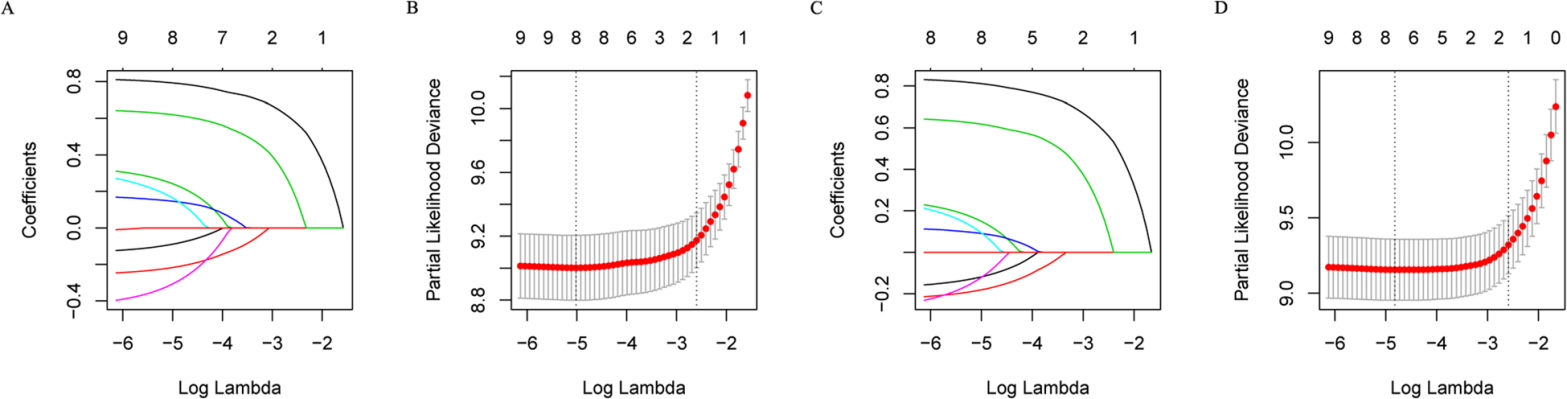
The LASSO regression used to select prognostic factors for OS and CSS; **a** LASSO coefficient profiles of 11 variables for OS; **b** LASSO Cox analysis identified 8 variables for OS; **c** LASSO coefficient profiles of 11 variables for CSS; **d** LASSO Cox analysis identified 8 variables for CSS. OS: overall survival; CSS: cancer-specific survival

**Fig. 2:**
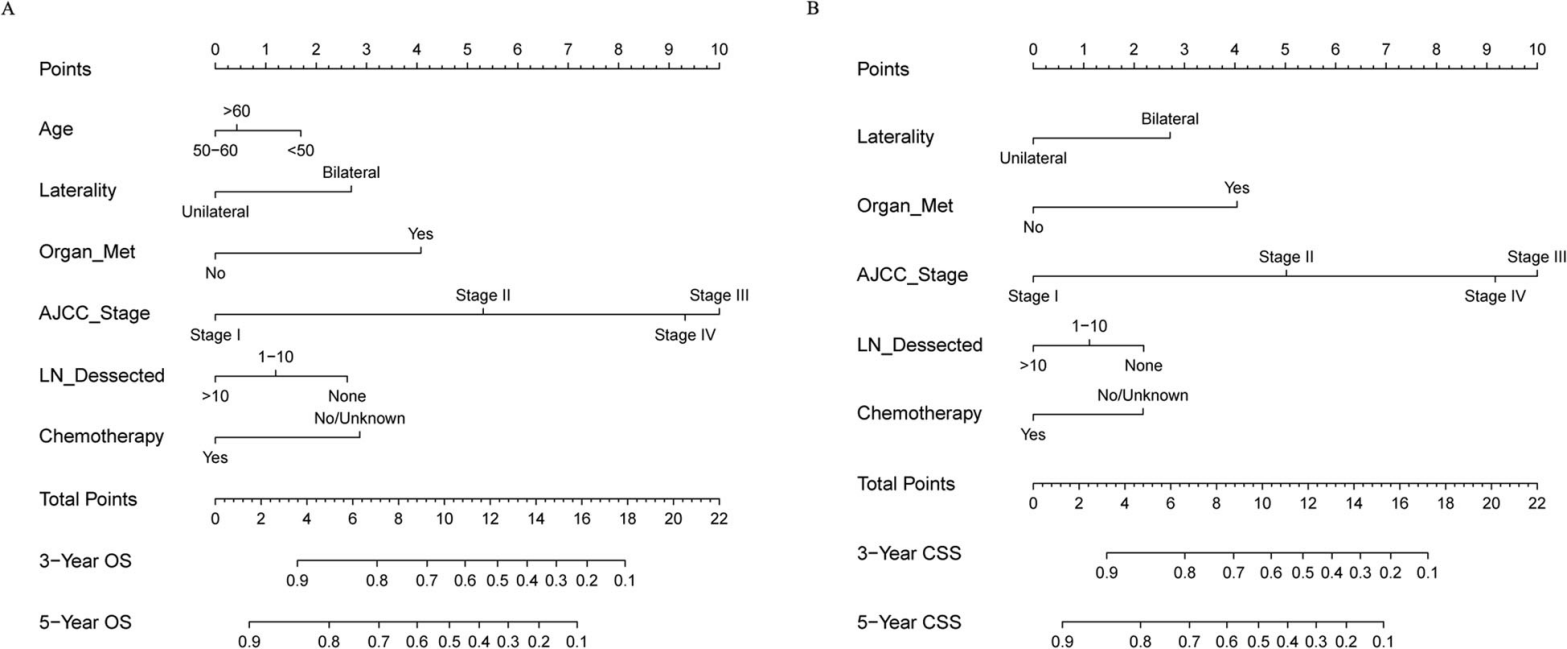
Predictive nomograms. **a** Nomogram for predicting 3- and 5-year OS; **b** Nomogram for predicting 3- and 5-year CSS. Met: metastasis; AJCC: American Joint Commission on Cancer; LN: lymph node; OS: overall survival; CSS: cancer-specific survival

In total, 11 variables were included in the analysis. According to the results of LASSO Cox regression analysis, age, laterality, stage, LN dissected, LN status, organ metastasis, radiotherapy and chemotherapy were identified for OS and CSS risk factors (Fig. 1). In the multivariate analysis of these 8 factors, age, laterality, stage, LN dissected, organ metastasis and chemotherapy were independently and significantly associated with OS, while laterality, stage, LN dissected, organ metastasis and chemotherapy were independently and significantly associated with CSS (Table 3). Based on the above, nomograms were constructed by incorporating the prognostic factors to predict 3- and 5-year OS and CSS (Fig. 2).

**Table 3:** Multivariate Cox analysis of the training cohort based on the results of lasso regression

	OS		CSS	
	HR (95% CI)	P	HR (95% CI)	P
Age, years				
< 50	Reference		Reference	
50–60	0.694 (0.494–0.974)	**0.035**	0.714 (0.501–1.018)	0.063
> 60	0.781 (0.556–1.098)	0.155	0.735 (0.511–1.057)	0.097
Laterality				
Unilateral	Reference		Reference	
Bilateral	1.789 (1.283–2.496)	**< 0.001**	1.792 (1.265–2.538)	**0.001**
AJCC Stage (7th)				
I	Reference		Reference	
II	3.039 (1.806–5.116)	**< 0.001**	3.035 (1.725–5.341)	**< 0.001**
III	8.683 (6.015–12.536)	**< 0.001**	9.168 (6.204–13.549)	**< 0.001**
IV	7.466 (4.318–12.909)	**< 0.001**	7.862 (4.392–14.073)	**< 0.001**
LN dissected, n				
No	Reference		Reference	
1–10	0.738 (0.517–1.054)	0.094	0.765 (0.524–1.118)	0.167
> 10	0.565 (0.403–0.794)	**< 0.001**	0.597 (0.416–0.856)	**0.005**
LN				
No dissection or Negative	Reference		Reference	
Positive	1.341 (0.884–2.035)	0.168	1.247 (0.794–1.958)	0.337
Organ Metastasis				
No	Reference		Reference	
Yes	2.326 (1.203–4.497)	**0.012**	2.256 (1.123–4.535)	**0.022**
Radiotherapy				
No/Unknown	Reference		Reference	
Yes	1.598 (0.776–3.287)	0.203	1.526 (0.705–3.301)	0.283
Chemotherapy				
No/Unknown	Reference		Reference	
Yes	0.531 (0.362–0.780)	**0.001**	0.615 (0.401–0.944)	**0.026**

### Nomogram validation

The C-indexes for the nomogram of OS and CSS in the training cohort were 0.802[95% confidence interval (CI) 0.773–0.831] and 0.802 (0.769–0.835), respectively, both of which were greater than the AJCC staging system [OS:0.775 (0.746–0.804); CSS: 0.781 (0.750–0.813)]. In the validation cohort, the C-indexes for the new model of OS and CSS [0.746 (0.691–0.801) and 0.770 (0.721–0.819), respectively] also presented superiority over the AJCC staging system [OS: 0.731 (0.684–0.778); CSS: 0.752 (0.707–0.791)].

**Fig. 3:**
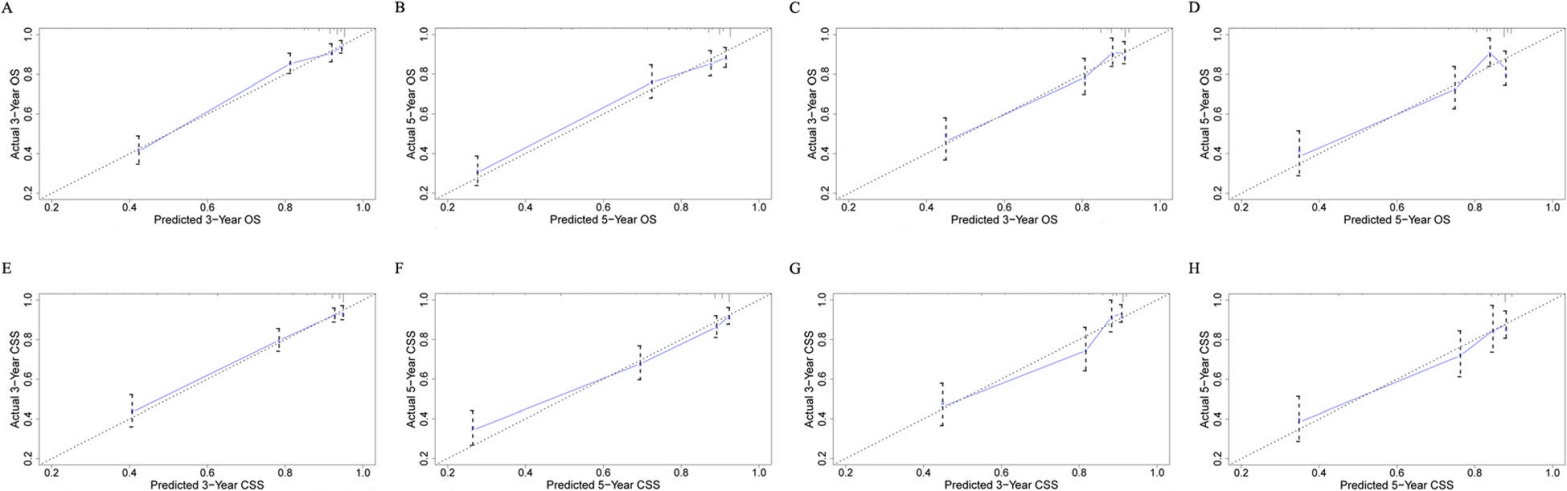
Calibration plots. **a** 3-year and (**b**) 5-year OS nomogram calibration plots for training cohort; **c** 3-year and (**d**) 5-year OS nomogram calibration plots for validation cohort; **e** 3-year and (**f**) 5-year CSS nomogram calibration plots for training cohort; **g** 3-year and (H) 5-year CSS nomogram calibration plots for validation cohort. OS: overall survival; CSS: cancer-specific survival

**Fig. 4:**
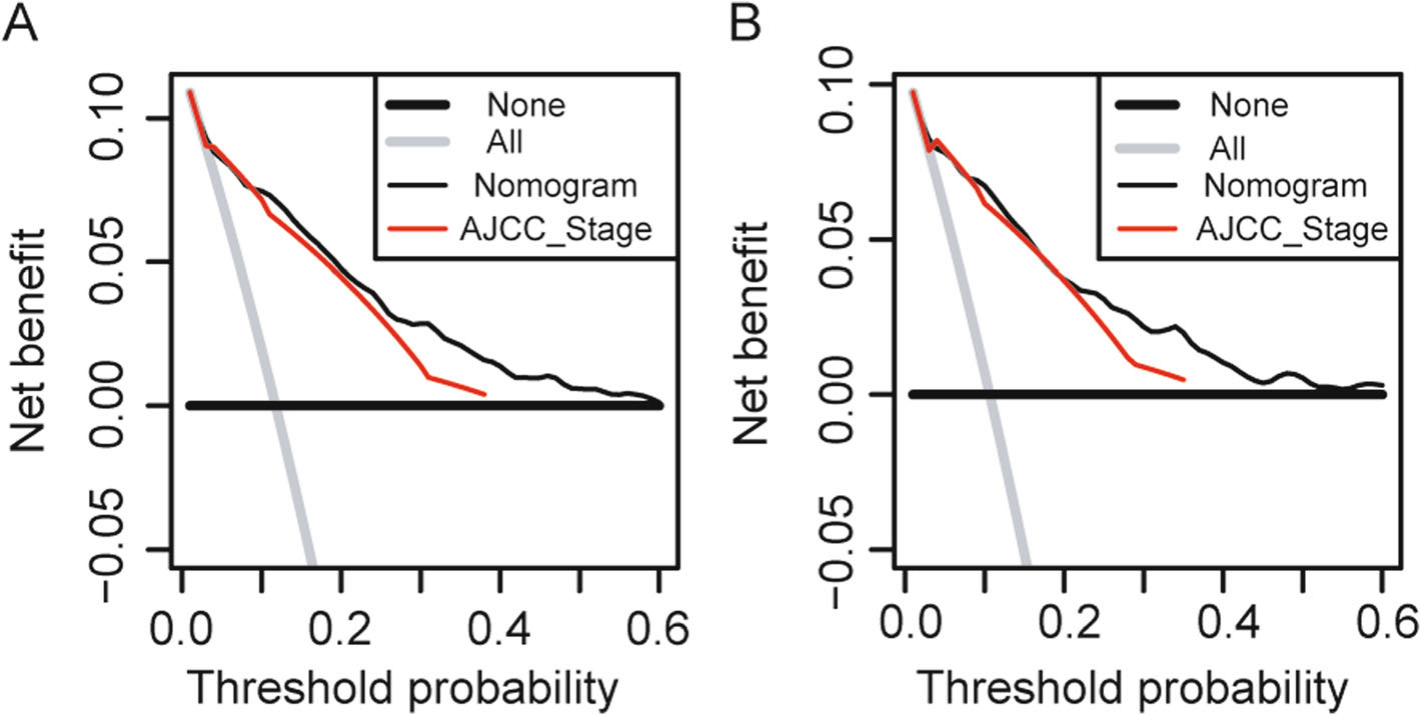
DCA curve of the nomogram and AJCC stage for (**a**) OS and (**b**) CSS. DCA: decision curve analysis; AJCC: American Joint Commission on Cancer; OS: overall survival; CSS: cancer-specific survival

The calibration curves indicated excellent agreement between the nomogram predicted and actual survival outcomes in the training and validation cohort (Fig. 3). DCA curves indicated that the nomogram models made favorable predictions and outperformed the AJCC staging system (Fig. 4).

### Risk stratification of OCCC patients

**Fig. 5:**
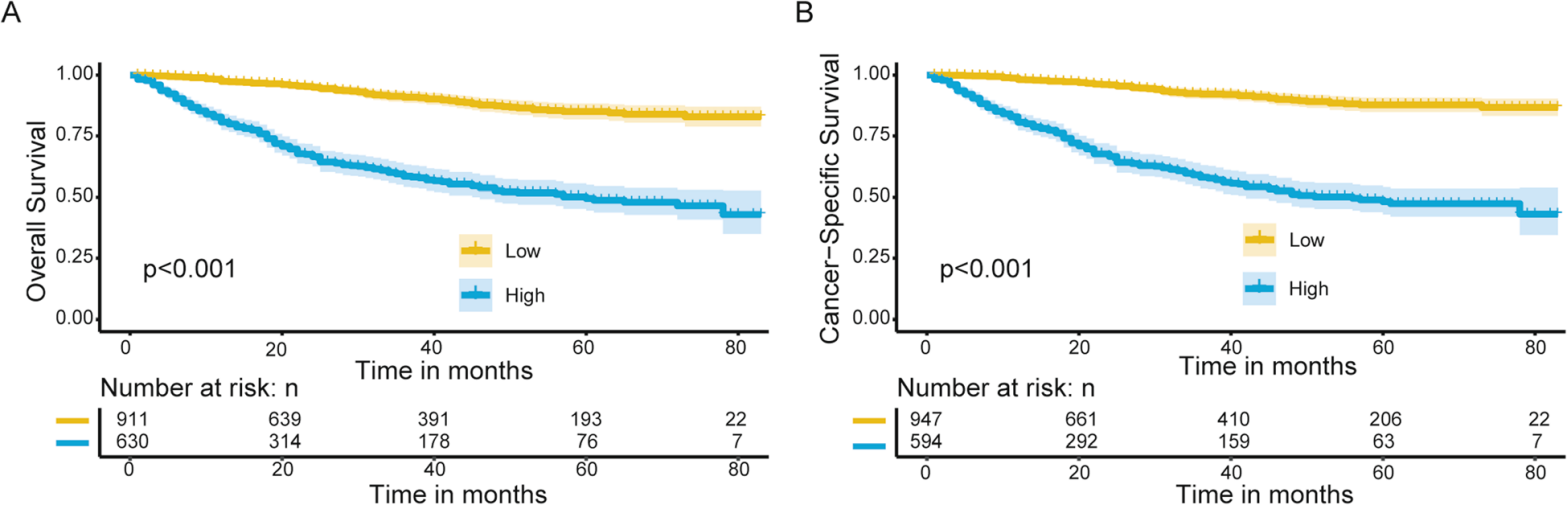
Kaplan-Meier curves. **a** Overall survival and (**b**) cancer-specific survival for patients stratified by the risk stratification system

The risk score of each variable was generated from the nomogram and the total scores were calculated for all the patients. The median risk score was 6 (range: 3–21) for OS and 4 (range: 2–19) for CSS. The whole cohort was divided into low- and high- risk subgroups based on the median risk score. According to the survival curves in Fig. 5, significant differences were observed between the low- and high -risk groups for both OS (*P* < 0.001) and CSS (*P <* 0.001), implying the nomogram’s outstanding ability for risk stratification.

## Discussion

In the current study, data of patients with OCCC who have undergone surgery in the SEER database were used for the analysis of risk factors. Nomograms were constructed to assess the 3- and 5-year CSS and OS based on the identified prognostic factors. Favorable discrimination and calibration were observed from C-index, calibration curves and DCA curves in both training and validation sets, indicating excellent performances of the nomograms. Moreover, risk scores generated from the nomograms were applied to successfully build a risk stratification system.

Our study identified six independent prognostic factors for OS: age, tumor laterality, organ metastasis, LN dissected, stage and chemotherapy. These factors also significantly impact CSS, except for age. Generally, patients at an older age are more likely to present worse survival outcomes due to lower immune response [11]. However, we observed that patients younger than 50 years tended to have poorer prognosis. One of the possible explanations may be the relatively conservative surgical mode for patients who wanted to preserve fertility. In addition to age, the relationship between other demographic characteristics (such as race) and prognosis was also explored.

Systematic lymphadenectomy was regarded as an important part of treatment guidelines for patients with EOC considering the prognostic value of LN status [12]. We observed that patients with more than 10 lymph nodes removed were associated with better prognosis. A 10-lymph node cutoff was defined as adequate lymphadenectomy according to the Gynecologic Oncology Group criteria. Several retrospective studies have demonstrated favorable survival outcomes of systematic lymphadenectomy on patients with early-stage OCCC [13–15]. In a combined exploratory analysis of three prospectively randomized phase III multicenter trials, Magazzino et al. reported that lymphadenectomy offered benefit to patients with advanced OC who received complete intraperitoneal debulking [16]. However, another study did not observe significant improvement in the survival of advanced OCCC patients with systematic retroperitoneal lymphadenectomy [17]. It is worth noting that LN status was not a prognostic factor in our study. Therefore, the role of lymphadenectomy on the whole cohort of OCCC patients required further investigation.

Chemotherapy is important in the management of EOC, especially for high-grade cases, while the role of chemotherapy for patients with OCCC remains controversial [18]. It was reported that the response rate of OCCC to conventional platinum was much lower than serous type in the first-line setting [19]. Published studies mainly focused on the performance of chemotherapy on early-stage cases [20–22]. In our study, we noted that chemotherapy was significantly associated with OS and CSS, implying its value in improving survival outcomes. Toru and his group carried out a randomized phase III trial (JGOG3017/GCIG Trial) to make a comparison between two chemotherapy regimens for OCCC. They demonstrated that irinotecan plus cisplatin and paclitaxel plus carboplatin were both well tolerated with no significant difference in survival benefit [23].

Several studies have reported better performance of the nomogram model than conventional staging systems and proposed it as a promising tool for prognosis evaluation [24–26]. Diao and his group developed a nomogram to predict OS in patients with inflammatory breast cancer, with a C-index of 0.738 (0.717–0.759, 24]. Zhou et al. generated nomograms to predict the CSS and OS for stage I–III colon cancer patients, achieving a C-index of 0.78 (0.77–0.80) for CSS and 0.74 (0.73–0.75) for OS [25]. Similarly, a prediction model was constructed for patients with non-small cell lung cancer, with a C-index of 0.674 (0.652–0.696, 26]. All of the above nomograms presented better discriminatory capacity than did the staging systems. Lack of some clinical information was the common limitation for these studies.

The nomograms developed in our study also presented better prediction capacity than AJCC 7th staging system. The nomogram model enables risk stratification of patients, thus facilitating personalized treatment plans and follow-up schedules. Considering the chemo-resistant feature of OCCC, efforts have been made to explore precision medicine based on molecular profiles, such as drugs targeting ARID1A-deficient OCCC patients [27]. It may be feasible to use a prediction model to select candidates for clinical trials.

It should be noted that there are several limitations in our study. First, detailed information about chemotherapy and radiotherapy as well as surgical procedures were unavailable. Data about the recurrence and reoperation were also unavailable in the SEER database. Second, selection bias was inevitable due to the study’s retrospective nature. Third, the nomogram model only received internal validation. External validation of cohorts from other countries and prospective randomized clinical trials are were necessary to confirm its performance.

## Conclusion

Nomograms with favorable capacity of prognosis assessment of 3- and 5-year OS and CSS for patients with initially diagnosed OCCC were constructed using data from a large-scale dataset. A risk stratification system was built based on risk scores generated from the nomograms. These nomograms may be useful to provide prognostic information in clinical work.

## Data Availability

The datasets used and/or analyzed during the current study are available from the publicly available SEER database.
